# Core competencies of healthcare professionals in Oman: Research and evidence‐based practice needs attention

**DOI:** 10.1002/nop2.1453

**Published:** 2022-11-02

**Authors:** Fatma Al Jabri, Tarja Kvist, Hannele Turunen

**Affiliations:** ^1^ Department of Nursing Science University of Eastern Finland Kuopio Finland

**Keywords:** clinical setting, core competence, healthcare professionals, nurses, Oman, physicians

## Abstract

**Aim:**

The aim of the study was to examine (1) the perceptions on core competencies of healthcare professionals working at clinical settings in Oman and (2) which demographic characteristics explain the overall core competency.

**Design:**

A cross‐sectional design.

**Methods:**

Healthcare Professional Core Competency Instrument, consisting of 11 sub‐scales with 81 items, was distributed to healthcare professionals (*n* = 1,543; 826 nurses and 717 physicians) who worked at primary, secondary and tertiary healthcare institutions. Descriptive statistics, t‐test, ANOVA and linear regression were used for data analysis.

**Results:**

Altogether 1,078 healthcare professionals (628 nurses and 450 physicians) responded representing 70% overall response rate. Healthcare professionals perceived their overall core competence as excellent, safety being the highest, and research and evidence‐based practice was the lowest. The multiple linear regression analysis revealed that ethnicity, gender and years of working experience were the characters that explained the overall core competence, where expatriate senior professionals reported higher competency levels compared with counterparts.

## 1 INTRODUCTION

Optimizing healthcare system continues to be on healthcare leaders' top agenda. Nevertheless, this can be challenging in view of escalating patients' expectations, increasing demand, advancing technological developments, worsening geopolitics, shortages in staff and increasing the number of errors (Gosling et al., 2019; Institute of Medicine [IOM], 2013). This issue is further magnified during times of pandemics, such as during COVID‐19, that require an unparalleled demand for the care of people in the wake of fear, stigma, misinformation and limitations on movement that disrupt the delivery of health care for all conditions (American Nurses Association [ANA], 2020; Ozdemir & Kerse, 2020). Such challenges can be exacerbated within the workforce if staff's competency is low in implementing appropriate measures despite adequate qualification and training (World Health Organization [WHO], 2020). These concerns create greater emphasis on the operational processes' efficiencies, integrated system optimisation and the competencies of professionals delivering care. The interplay of nurses and physicians – as the major constituents of the healthcare system – play a pivotal role in the integrated healthcare mission (Lalloo et al., 2017; Lombarts et al., 2014).

While nurses' and physicians' technical and functional competencies continue to evolve in line with the evidence‐based practices (EBP), technological developments and rising expectations, there is a great requirement to measure the level of the “core” competencies that are fundamental in assuring an extended value‐focused, efficiency‐based and transformable healthcare system (Flinkman et al., 2017; IOM, 2013). Professionals' core competency has been proposed as one of the factors that impact patient safety and job satisfaction (Albarqouni et al., 2018). These core competencies define a cluster of attributes of knowledge, skills and attitudes that allow the healthcare professionals (HCPs) to perform tasks following acceptable delivery standards (Albarqouni et al., 2018; IOM, 2013; Tromp et al., 2012).

This study aimed to examine the core competencies of both nurses and physicians working at clinical settings in Oman. This investigation is based on an adopted and extensive instrument that consisted of 11 core competencies while the available literature focuses predominantly on the core competencies of either an individual profession (nurses or physicians) (Albarqouni et al., 2018; Faraji et al., 2019; Keykha et al., 2016; Mirlashari et al., 2016; Wei et al., 2018) or covers selective competence sub‐scales (Al Lawati et al., 2019; Al‐Busaidi et al., 2019; Al‐Saadi et al., 2019; Alvarez et al., 2021; Fu et al., 2020; Kanerva et al., 2015; Kantanen et al., 2017; Khezri & Abdekhoda, 2019; Mert Boğa et al., 2020; Mohamed et al., 2020; Rouse & Al‐Maqbali, 2014; Suhonen et al., 2021; Ylitörmänen et al., 2019).

This shall add to the body of knowledge an insight for cross‐integration and a reflective measure of the overall quality care delivery system. This will also determine the training and upskilling requirements for the healthcare team (Ozdemir & Kerse, 2020; WHO, 2020).

## 2 BACKGROUND

The healthcare system continues to endure transformation to improve delivery quality, reduce associated costs and increase stakeholder experience. Calls for such transformation continue to take centre stage at times of unparalleled challenges and pandemics, such as those of COVID‐19 (ANA, 2020; WHO, 2020). This transformation requires HCPs to continue upgrading their “core” competencies in line with the evolving relevant standards and certification programs (Accreditation Council for Graduate Medical Education (ACGME), 2020; American Nurses Credentialing Center [ANCC], 2020; European Commission, 2020; Singapore Nursing Board, 2018). These standards and programs tabulate the minimum core competencies that include (1) quality improvement, (2) professionalism, (3) communication, (4) teamwork and collaboration, (5) patient‐centred care, (6) research and EBP, (7) leadership and management, (8) personal and professional development, (9) ethical and legal practice, (10) safety and (11) health information technology. Core competencies are defined as the skills, values, attitudes and beliefs that the organization stands for and that all HCPs must uphold and demonstrate every day (Albarqouni et al., 2018).

Quality improvement refers to the use of historical and current performance to derive best practices for improving the healthcare delivery system (Gosling et al., 2019). This competence was measured among HCPs and patients in Ghana (Abuosi, 2015) and Germany (Willems & Ingerfurth, 2018) in which a significant difference in the overall perception of quality of care between patients and HCPs was reported in both studies.

Professionalism entails the mechanics of healthcare delivery in reference to the best humanistic, moral, ethical, regulatory and legal practices (Lombarts et al., 2014). Lombarts et al. (2014) measured this competence among physicians and nurses in European hospitals which concluded that physicians and nurses display equally high professionals' attitude.

Communication enables effective interaction between HCPs and patients to increase healthcare efficiency and to upgrade quality services and safe clinical practice (Sheldon & Hilaire, 2015). The role of this competence was investigated among nursing professionals in Oman (Rouse & Al‐Maqbali, 2014), Finland (Kanerva et al., 2015; Syyrilä et al., 2020) and Malaysia (Amudha et al., 2018). These studies highlighted the direct role of communication skills in developing patient safety practices and strategies.

Teamwork and collaboration are essential competencies that urge HCPs to exhibit a shared vision, mutual understanding, team bonding and integrated delivery (Ylitörmänen et al., 2019). Studies in Finland and Norway (Ylitörmänen et al., 2019), Egypt (El Sayed & Sleem, 2011) and Australia (Suryanto et al., 2016) indicated positive attitudes from nurses and physicians towards teamwork and collaboration.

Patient‐centred care (PCC) relates to the HCPs' ability to realize patients' expectations, preferences and values and to work together with patients to deliver compassionate, safe and effective care (Delaney, 2018). Suhonen et al. (2021) assessed the level of PCC mong Finnish nurses and indicated that PCC was rated as good.

Research and EBP competencies are fundamental for the continuous improvement of healthcare services and delivery processes based on learnings and best practices (Paudel et al., 2018). This competence was measured among HCPs in Oman (Al‐Busaidi et al., 2019; Ammouri et al., 2014), Jordan (AbuRuz et al., 2017), China (Fu et al., 2020) and Spain (Alvarez et al., 2021). Those studies showed that HCPs had positive attitudes towards research and EBP and they considered it as a key component to improve practice, nevertheless the levels of their perceived skills in this dimension were reported as low to moderate.

Leadership and management are another overarching competence that assures performance and drives organizational growth (Sfantou et al., 2017). Kantanen et al. (2017) conducted a study among nursing leaders to describe leadership and management competencies at healthcare settings in Finland – which indicated that leaders had a good leadership and management competence. Personal and professional development competence relates to the continuous learning and education and the determination towards positive change and criticism in accordance with professional standards (Manley et al., 2018).

Healthcare professionals have to maintain high levels of ethical and legal competence at all times in accordance with the code of ethics, code of conduct and associated laws and regulations (Pozgar, 2016). The participants' views of ethical and legal aspects were measured at healthcare settings in Oman (Al‐Saadi et al., 2019) that showed high awareness of the importance of patients' rights among nurses and physicians but with low actual adherence levels in practice. Other studies in Australia (Lamont et al., 2019) and Egypt (Aly et al., 2020) found that knowledge of patients' rights and associated decision‐making capacity was insufficient and had a negative influence on the occurrence of medical errors.

Safety is another competence that refers to HCPs' ability to prevent risks and adverse effects on patients through delivery competence and system effectiveness (Lavin et al., 2015). Al‐Mandhari et al. (2018) revealed that implementation of Patient Safety Friendly Hospital Initiative (PSFHI) in selected hospitals in Oman had successful outcomes in improving patient's safety. In another study in Oman, Al Lawati et al. (2019) evaluated this implementation in primary care institutions in Oman in which HCPs rated patient safety as very good to excellent. Other studies nevertheless indicated low perception of patient safety practice among healthcare providers in Ethiopia (Gizaw et al., 2018) and Egypt (Eldeeb et al., 2016).

Health information technology (HIT) resembles HCPs' ability to use information technology in the way that is most appropriate for improving healthcare quality and effectiveness (Lavin et al., 2015). This competence was evaluated by Khezri and Abdekhoda (2019) among 205 nurses working at Tabriz University of Medical Sciences' hospitals. This study stated that nurses have a good level of informatics skills (62.98%) and knowledge (59%).

This study's purposes were to examine (1) the perceptions on core competencies of HCPs working at clinical settings in Oman and (2) which demographic characteristics explain the overall core competency. This will add to the existing body of knowledge a comprehensive line of sight of the level of core competencies for both nurses and physicians.

## 3 THE STUDY

### 3.1 Study context

This study was conducted in Oman – a country located in the south‐eastern Arabian Peninsula with an area of 309,500 square kilometres. Oman has a population of around 4.6 million (National Centre for Statistics and Information [NCSI], 2020). Oman's healthcare system comprises both public and private sectors. The public sector is further sub‐divided into the Ministry of Health (MOH) and the non‐MOH. The MOH comprises 50 hospitals and 211 healthcare centres (Department of Health Information and Statistics, 2019).

The non‐MOH comprises hospitals that serve the employees and their families who belong to a certain sector such as Ministry of Defence Hospital, and Royal Oman Police Hospital. The private sector includes private hospitals, general and specialized clinics (Department of Health Information and Statistics, 2019). MOH hospitals provide the majority of health services. Oman's government covers all medical expenses for its citizens.

### 3.2 Design

This study used a cross‐sectional study design. Study reporting followed STROBE checklist (see Table A1 in Appendix A).

### 3.3 Method

#### 3.3.1 Participants and setting

This study targeting all nurses and physicians working at all healthcare institutions (primary, secondary and tertiary) under the MOH in Oman with at least 1 year of experience; excluding students and interns. The total target group was 20,966 (14,433 nurses and 6,533 physicians) (Department of Health Information and Statistics, 2019). Proportional stratified sampling was used to recruit different professions (nurses and physicians) working at primary, secondary and tertiary healthcare institutions. The total sample size was 1,543 (826 nurses and 717 physicians). A website calculator called “Raosoft” was used to calculate the sample size wherein the margin of error and confidence level were taken as 5% and 95%, respectively. The resulting power analysis was 374 nurses and 363 physicians (Meysamie et al., 2014).

#### 3.3.2 Instrument

The Healthcare Professional Core Competency Instrument (HPCCI) was used for data collection. The HPCCI was adopted from the existing valid and reliable tools and the permission to use the tools was granted by their developers (see Table A2 in Appendix B). The instrument was in English and was piloted through convenient sampling of 56 (36 nurses and 20 physicians) HCPs at tertiary hospital in Oman. This sample size was calculated based on Raosoft calculation of sample size. Cronbach's alpha coefficient of the piloted study ranged from 0.697 to 0.980 for the 11 core competency sub‐scales. These values were considered acceptable and a good level of internal consistency in healthcare research projects (Tavakol & Dennick, 2011). The newly adopted instrument was fit for both nurses and physicians and can be applied in different settings. Based on the pilot, there were no changes in the tool.

The questionnaire had two parts. The first part had demographic characteristics: profession, age, gender, ethnicity, position, years of working experience, education, institution level and working area. The second part had 11 sub‐scales with 81 items to assess the participants' self‐rated core competencies as follows: quality improvement (four items such as “Anticipate the variability of clinical practice and acts proactively in the implementation of interventions that ensure quality”), professionalism (six items such as “Treat others respectfully even if they hold different opinions”), communication (10 items such as “Promote an environment to facilitate communication”), team work and collaboration (seven items such as “Collaborate with members of the multidisciplinary team”), patient‐centred care (10 items such as “Involve the patient and family in the planning and implementation of care”), research and EBP (eight items such as “Assess current clinical practice, on an individual and systemic level based on the latest research findings”), leadership and management (10 items such as “Delegate responsibility for care based on assessment of abilities of individuals”), personal and professional development (eight items such as “Identify your own professional development needs by reflecting on practice”), ethical and legal practice (eight items such as “Carry out clinical practice according to legal requirements and organizational policy”), safety (seven items such as “Report and response to an error”) and health information technology (three items such as “Use information and communication technologies in the delivery of patient/client care”). This study's total Cronbach's alpha coefficient was 0.917 and ranged from 0.792 to 0.926 for the 11 core competency sub‐scales (Table 2). A five‐point Likert scale (1: Never, 2: Rarely, 3: Sometimes, 4: Very Often, 5: Always) was used to answer the second part of questionnaire.

#### 3.3.3 Data collection

Questionnaires along with the fact sheets and consent forms were handed over to the research assistants, who were allocated as focal points for the study in each institution. Questionnaires were distributed for nurses and physicians who provided written informed consent in all working areas over a period of 1 – month – by end of 2018 and the beginning of 2019. Each completed questionnaire was inserted in an envelope at locked boxes allocated in each unit. During that period, the research assistants in each institution verbally announced reminders about the study to the HCPs.

### 3.4 Analysis

Data were analysed with the Statistical Package for the Social Sciences Computer Program (SPSS version 27.0) using descriptive analysis (frequency, percentage, mean value, standard deviation). The overall core competence variable was a computed variable of 11 core competency sub‐scales with 81 items. ANOVA, *t*‐test and linear regression were utilized to explain the factors that contribute to the overall core competence level. Age was recorded into three groups: <30 years, 30–40 years and >40 years and working experiences were categorized as <8 years, 8–15 years and >15 years.

To aid the presentation of the results in this study, researchers have set a mean value of 4.0 or more to indicate an “excellent” core competence level. This is based on best practices in the literature and magnet hospital assessment scales, where four is defined as meeting Magnet standards (ANCC, 2017).

## 4 RESULTS

### 4.1 Healthcare professionals' demographic characteristics

The overall response rate was 70% (1,078 of 1,543 overall target). This response constitutes of 58.3% nurses and 41.7% physicians (Table 1). Most of the respondents fell within 30–40 age group and were females with a share contribution of 62% and 71%, respectively. The response of Omani staff was 11% higher compared with that of expatriates. Around 65% of respondent HCPs worked at bed side, followed by those who had dual roles, that is clinician and management. Around two‐thirds of respondents had between 8–15 years of working experience. The majority of nurses and physicians had a diploma (70%) and specialist (56%) degrees, respectively, as an educational background. More than half (52%) of the HCPs worked in tertiary hospitals seconded by primary healthcare centres.

**TABLE 1:** Healthcare professionals' characteristics (*n* = 1,078)

Demographic characteristics		*n*	%
Profession	Nurses	628	58.3
	Physicians	450	41.7
Age in (years)	<30	148	15.1
	30–40	609	62.0
	>40	225	22.9
Gender	Female	762	71.0
	Male	311	29.0
Ethnicity	Omani	592	55.3
	Non‐Omani	478	44.7
Position	Clinician	608	64.8
	Management	45	4.8
	Both	285	30.4
Working experiences in (years)	<8	268	26.0
	8–15	482	46.8
	>15	279	27.1
Education for nurses	Diploma	436	69.5
	Bachelor	180	28.7
	Master	11	1.8
Education for physicians	Resident	152	37.0
	Specialist	231	56.2
	Adjunct	7	1.7
	Docent	9	2.2
	Professor	12	2.9
Level of care	Tertiary	563	52.2
	Secondary	127	11.8
	Primary healthcare	388	36.0
Working area	Medical	173	16.2
	Surgical	157	14.7
	Paediatrics	106	10.0
	Obstetrics and gynaecology	122	11.5
	Critical care	156	14.6
	Outpatient clinics	235	22.1
	Others	116	10.9

### 4.2 Core competence level of healthcare professionals

Table 2 presents the mean scores of 11 core competency sub‐scales showing that nine of them were above the target level (≥4.0). Safety sub‐scale (M = 4.545; *SD* = 0.484) was given the highest rating followed by ethical and legal practice (M = 4.460; *SD* = 0.511). The scores of quality improvement and research and EBP were under the target level, with 3.945 (*SD* = 0.666) and 3.720 (*SD* = 0.780), respectively, reflecting good levels rather than excellent. The overall mean (M) score and standard deviation (*SD*) for all 11 sub‐scales stood at excellent level (M = 4.262; *SD* = 0.423).

**TABLE 2:** Core competencies of HCPs

Sub‐scale	M	*SD*	Cronbach's alpha
Safety	4.545	0.484	0.894
Ethical and Legal Practice	4.460	0.511	0.862
Teamwork and Collaboration	4.370	0.531	0.873
Health Information Technology	4.330	0.618	0.876
Professionalism	4.340	0.511	0.795
Patient‐Centred Care	4.325	0.514	0.885
Communication	4.315	0.484	0.837
Personal and Professional Development	4.285	0.559	0.905
Leadership and Management	4.245	0.569	0.913
Quality Improvement	3.945	0.666	0.792
Research and EBP	3.720	0.780	0.926
Overall core competence	4.262	0.423	0.917

Abbreviations: M, mean; *SD*, standard deviation.

### 4.3 Relationship between HCPs' demographic characteristics and overall core competency level

There were no significant differences in nurses' (M = 4.240; *SD* = 0.421) and physicians' (M = 4.280; *SD* = 0.425) assessments concerning the overall core competence level (Table 3). HCPs aged 40 years or older rated themselves more competent (M = 4.370; *SD* = 0.432; *p* < 0.001) compared with younger age groups. The Omani staff scored their overall core competence lower than expatriates (M = 4.180; *SD* = 0.427; *p* = 0.038). HCPs with more than 15 years of work experience resembled a higher overall core competence level (M = 4.330; *SD* = 0.434; *p* < 0.001) relative to professionals with lesser experience. HCPs working in the secondary healthcare level (M = 4.310; *SD* = 0.440; *p* = 0.011) reported slightly higher overall core competence level compared with those working in tertiary or primary healthcare institutions. Participants from a critical care unit credited themselves with overall core competence (M = 4.360; *SD* = 0.375; *p* < 0.001) in comparison with participants from other working areas.

**TABLE 3:** Relationship between HCPs' demographic characteristics and overall core competency level (*n* = 1,078)

Demographic characteristics	Overall Core Competency Level		
	M	*SD*	*p*
Profession			0.822
Nurses	4.240	0.421	
Physicians	4.280	0.425	
Age in (years)			<0.001
<30	4.110	0.437	
30–40	4.240	0.408	
>40	4.370	0.432	
Gender			0.312
Female	4.220	0.425	
Male	4.350	0.402	
Ethnicity			0.038
Omani	4.180	0.427	
Non‐Omani	4.360	0.397	
Position			0.355
Clinician	4.260	0.419	
Management	4.180	0.462	
Both	4.240	0.414	
Working experiences in (years)			<0.001
<8	4.130	0.426	
8–15	4.280	0.402	
>15	4.330	0.434	
Level of care			0.011
Tertiary	4.280	0.425	
Secondary	4.310	0.440	
Primary	4.210	0.410	
Working area			<0.001
Medical	4.270	0.379	
Surgical	4.320	0.470	
Paediatrics	4.220	0.394	
Obstetrics and gynaecology	4.290	0.474	
Critical care	4.360	0.375	
Outpatient clinics	4.170	0.413	
Others	4.190	0.435	

Abbreviations: M, mean; *SD*, standard deviation; *p*, *p*‐value.

### 4.4 Demographic characteristics explaining the overall core competency level

A multiple linear regression analysis was performed to examine which demographic characteristics were explaining the level of HCPs' overall core competency. The adjusted *R* square was 0.071, which means that 7% of overall core competency is explained by the combined demographic characteristics. This analysis shows that ethnicity had the strongest relationship with the overall core competence level. The standardized regression coefficients were significantly higher in non‐Omani participants compared with Omani (β = 0.158; *p* < 0.001; Table 4).

**TABLE 4:** Multiple linear regression analysis assessing whether demographic characteristics explain the levels of core competence (*n* = 1,078)

Dependent variable: overall core competency level							
Independent variable	B	SE	β	*t*	*p*	95% CI	
Constant	4.173	0.053		78.163	<0.001	4.069	4.278
Profession: Physician (ref: Nurse)	−0.027	0.031	−0.032	−0.884	0.377	−0.088	0.033
Age in (years)							
30–40 years (ref: <30 Years)	0.006	0.036	0.007	0.172	0.863	−0.064	0.076
>40 Years	0.009	0.050	0.009	0.181	0.857	−0.089	0.107
Gender: Female (ref: Male)	−0.085	0.034	−0.091	−2.506	0.012	−0.151	0.018
Ethnicity: Non‐Omani (ref: Omani)	0.134	0.029	0.158	4.643	<0.001	0.077	0.191
Position							
Management (ref: Clinician)	−0.084	0.065	−0.040	−1.293	0.196	−0.210	0.043
Clinician and management	−0.005	0.029	−0.005	−0.168	0.867	−0.062	0.052
Working experiences in (years)							
8–15 Years (ref: <8 Years)	0.105	0.034	0.123	3.123	0.002	0.039	0.171
>15 Years	0.132	0.042	0.137	3.127	0.002	0.049	0.215
Level of Care							
Secondary (ref: Tertiary)	0.057	0.042	0.043	1.365	0.173	−0.025	0.138
Primary health care	0.003	0.034	0.003	0.083	0.934	−0.064	0.069
Working Area							
Surgical (ref: Medical)	0.066	0.046	0.055	1.441	0.150	−0.024	0.157
Paediatrics	−0.011	0.051	−0.007	−0.210	0.833	−0.110	0.089
Obstetrics and gynaecology	0.043	0.050	0.032	0.856	0.392	−0.055	0.141
Critical care	0.068	0.046	0.056	1.473	0.141	−0.022	0.158
Outpatient clinics	−0.067	0.043	−0.066	−1.555	0.120	−0.152	0.018
Other units	−0.027	0.049	−0.020	−0.559	0.576	−0.124	0.069
F	5.865				< 0.001		
*R* ^2^	0.086						
Adjusted *R* ^2^	0.071						

Abbreviations: β, standardized regression coefficient; B, partial regression coefficient; CI, confidence interval; *p*, *p*‐value; SE, standard error; *t*, t‐statistics for the coefficient.

The results also revealed that HCPs who have worked for >15 years (β = 0.137; *p* < 0.010) and 8–15 years of experience (β = 0.123; *p* < 0.010), rated the overall competency level significantly higher than staff who were working for less than 8 years. Additionally, the results showed that female staff (β = −0.091; *p* < 0.050) scored the overall competency level significantly lower than male staff (Table 4).

## 5 DISCUSSIONS

### 5.1 Perspectives on HCPs' self‐rated core competencies

Excellent core competence levels – in reference to Magnet standards (ANCC, 2017) – were measured in 9 out of 11 sub‐scales, namely safety, ethical and legal practice, teamwork and collaboration, health information technology, professionalism, patient‐centred care, communication, personal and professional development and leadership and management. While quality improvement and research and EBP sub‐scales were under the target level of Magnet standard.

Healthcare professionals across Oman rated safety as the highest competence indicating HCPs feel confident about their skills, knowledge and attitude towards patient safety. Oman takes patient safety very seriously and is already showing regional leadership in the Eastern Mediterranean Region by adopting the PSFHI to improve the safety of healthcare in public and private hospitals nationwide (Al‐Mandhari et al., 2018). It is therefore not surprising to see that this result coincides with the findings of Al‐Mandhari et al. (2016) and Al Lawati et al. (2019) who reported patient safety competence rating (based on self‐assessment) for HCPs as very good to excellent. Al‐Mandhari et al. (2016) stated that HCPs in Oman demonstrated awareness and implementation of the WHO's nine “Life‐Saving Patient Safety Solutions”.

Similar international case studies done in Saudi Arabia (Alshammari et al., 2019) and South Korea (Jang & Lee, 2017) showed mean values lower than that of Oman with 3.560 and 4.110, respectively.

Research and EBP was rated under the target level indicating its integration in clinical practice is still a challenge in Oman – or a potential “jack‐up” opportunity, as some studies have shown that research and EBP was a direct reason behind 28% of the improvement in patient outcomes when clinical care was based on evidence rather than traditional practices (Melnyk et al., 2012). This finding was supported by other studies conducted in Oman (Al‐Busaidi et al., 2019; Ammouri et al., 2014), China (Hong & Chen, 2019) and Nepal (Paudel et al., 2018) which described the HCPs' practices, attitudes, knowledge/skills and perceived barriers in relation to EBP. These studies showed that while the attitudes of HCPs towards research and EBP were positive, their knowledge and implementation skills were low to moderate. These studies perceived that insufficient time for research and insufficient resources to change practice were barriers to developing the EBP among nurses and physicians.

### 5.2 Demographic characteristics explaining the level of overall core competency

There was a strong relationship between overall core competency and demographic characteristics (age, ethnicity, working experience, institution level of care and working area). The overall core competency level proportionately increased with more age and working experience. This trend was in line with reported observations of Adib‐Hajbaghery and Eshraghi‐arani (2018), and Rizany et al. (2018), while another study reported no significant differences with age (Faraji et al., 2019).

This relationship was further explored with the multiple linear regression analysis of the combined demographic characteristics. This analysis showed that gender, ethnicity and working experience were explained nurses' and physicians' overall core competency. Ethnicity was the main contributing character of the overall core competency level wherein expatriate professionals reported a higher competency level compared to their Omanis counterpart. This finding was similar to a study conducted among nurses and physicians in Oman by Al‐Saadi et al. (2019) which aimed to determine the extent of awareness and adherence to patients' rights, and it found that Non‐Omani staff were more aware (OR = 1.696; *p* = 0.032) and adherent (OR = 2.769; *p* = <0.001) to patient rights than Omani staff. The available literature does not report the underpinning causes for this; nevertheless it may be that some of the expatriates had received additional and/or specialized education/qualification on patients' rights in their previous career paths.

The multiple regression analysis revealed that gender also was a factor that contributed to the overall core competency, where male staff evaluated themselves as having a slightly higher amount of competence compared with female staff. This study is supported by Wangensteen et al. (2012), who stated male nurses assessed their competence higher compared with female nurses' self‐assessment. This study is nevertheless contradicted with another self‐reported study conducted by Adib‐Hajbaghery and Eshraghi‐arani (2018), who believed that clinical competency was a skill that all female and male nurses should have.

This study also shows that the self‐rated overall core competency was higher for staff with more than 15 years of experience compared to those with less working experience. The finding was in line with the results of Chang et al.'s (2011) and Rizany et al.'s (2018) studies, but inconsistent with Faraji et al.'s (2019) findings.

Furthermore, this study presents that staff working at tertiary and secondary institutions rated their competence higher than those at primary healthcare centres. This might be related to the highly specialized facilities, greater expertise and knowledge sharing and more exposure to critical cases. This observation was also seen by Abdulhadi et al. (2007) who reported less patient satisfaction with patient‐provider interactions in primary healthcare in Oman. Another study conducted in China confirmed the patient preference to use secondary and tertiary hospitals than primary health centres due to the services and quality of care provided in the hospitals setting (Hu et al., 2016; Li et al., 2020).

The working area also associated with the HCP's overall core competency level. Staff who worked in the critical care units were more competent than other providers working in other units. This result matches Lakanmaa et al.'s (2015) findings, which stated that intensive care unit (ICU) staff rated their competencies in a higher level. This may be due to the high knowledge and technical skills required in the ICUs (Chang et al., 2011).

### 5.3 Clinical implication

The subject of core competence continues to take top priority in Oman in line with the global paradigm shifts from volume to value as well as due to the sharply increasing expectations for ultra‐high‐care delivery (Undersecretariat for Planning Affairs, 2014).

This research has the following important implication dimensions:

1. The excellent stance of the core competencies of HCPs in Oman using a comprehensive adopted instrument provide a good indicator of the overall healthcare system in Oman. The sustainability of such excellent levels could be the focus of the policy and the technical leadership at the Ministry of Health in Oman.
2. The findings of the regression analysis can provide good insights for cross‐learning, upskilling and wider capacity building through establishing seamless avenues for younger professionals to learn from expatriates and experienced professionals.
3. Implementation of research and practice EBP is a key competence that require further development. This competence can impact the capacity for renewal of healthcare institutions in Oman. The capacity for renewal is a measure of the readiness of an organization to be fully engaged, agile and futuristic to position itself in reference to the internal and external environments and transformations (Delaney, 2016; Salmond & Echevarria, 2017; WHO, 2017).

These implications directly impact the human resources at the Ministry of Health in Oman. Human resources constitute an important pillar of the health system in Oman, not only because they form more than 73% of health expenditure but also because the quality of healthcare depends to a large extent on the quality of the workers (NCSI, 2020).

These implications will also provide important insights to the global community towards improving quality of care and patient safety from a core competence perspective.

### 5.4 Strengths and limitations

This study used HPCCI instrument adopted from valid and reliable tools that was developed carefully and reviewed by experts in the nursing field and piloted in the healthcare setting. The response rate of this study was good (70%).

The HPCCI scale was nevertheless based on a self‐assessment method that could influence the study's results. This suggests that HCPs' competence could be further verified through a complementing research work that utilizes observation‐based assessments and/or utilization of accredited competence consultants. It would also be very interesting to see how patients – as healthcare users – perceive such competence through the lens of healthcare quality and delivery.

## 6 CONCLUSIONS

Assessing HCPs' core competence is important in the journey of healthcare transformation. This study has demonstrated that the self‐rated overall core competency level of HCPs (nurses and physicians) in Oman stand at an excellent level. This sets a good scene for healthcare leaders in Oman to drive transformation within the sector with great confidence. Nevertheless, the study revealed that research and EBP should be further enabled, resources skills/upskilled and outcomes introduced into practice.

Gender, ethnicity and working experience explained nurses' and physicians' overall core competencies. A subsequent research work is recommended to explore how the healthcare system can address these factors and may capitalize on possible opportunities such as the transfer of knowledge within the workplace and stimulation of cross learning. Developing HCPs' core competencies remain an important impetus to strengthen the human resource capabilities and to sustain a high level of quality patient outcomes.

## AUTHOR CONTRIBUTIONS

All authors listed meet the authorship criteria according to the latest guidelines of the International Council of Medical Journal Editors and that all authors are in agreement with the manuscript. All authors designed this study. FA conducted data collection. All authors analysed and interpreted the data. FA drafted the manuscript, and all authors contributed substantially to its revision. FA takes responsibility for the manuscript as a whole.

## FUNDING INFORMATION

The first author received a grant from Ministry of Health, Oman.

## CONFLICT OF INTEREST

No conflicts of interest have been declared by the authors.

## ETHICAL APPROVAL

An ethical statement was obtained from the University of Eastern Finland Committee on Research Ethics (Statement 16/2018). In addition, an ethical statement including permission to conduct the study in the hospitals was obtained from the MOH in Oman (Proposal ID: MOH/CSR/18/XXXX). All data were treated confidentially, and the participants were allowed to withdraw at any time point.

## Data Availability

The data that support the findings of this study are available on request only from the corresponding author. The data are not publicly shared due to privacy and ethically restrictions.

## APPENDIX A

**TABLE A1:** STROBE statement—checklist of core competencies of healthcare professionals in Oman: Research and evidence‐based practice needs attention

	Item No	Recommendation
Title and abstract	1	(*a*) Indicate the study's design with a commonly used term in the title or the abstract **[1]**
		(*b*) Provide in the abstract an informative and balanced summary of what was done and what was found **[1]**
Introduction		
Background/rationale	2	Explain the scientific background and rationale for the investigation being reported **[1–5]**
Objectives	3	State specific objectives, including any prespecified hypotheses **[5]**
Methods		
Study design	4	Present key elements of study design early in the paper **[5]**
Setting	5	Describe the setting, locations, and relevant dates, including periods of recruitment, exposure, follow‐up and data collection **[6]**
Participants	6	(*a*) *Cohort study*—Give the eligibility criteria, and the sources and methods of selection of participants. Describe methods of follow‐up **[N/A]** *Case–control study*—Give the eligibility criteria, and the sources and methods of case ascertainment and control selection. Give the rationale for the choice of cases and controls **[N/A]** *Cross‐sectional study*—Give the eligibility criteria and the sources and methods of selection of participants **[6]**
		(*b*) *Cohort study*—For matched studies, give matching criteria and number of exposed and unexposed **[N/A]** *Case–control study*—For matched studies, give matching criteria and the number of controls per case **[N/A]**
Variables	7	Clearly define all outcomes, exposures, predictors, potential confounders, and effect modifiers. Give diagnostic criteria, if applicable **[N/A]**
Data sources/measurement	8^a^	For each variable of interest, give sources of data and details of methods of assessment (measurement). Describe comparability of assessment methods if there is more than one group **[6]**
Bias	9	Describe any efforts to address potential sources of bias **[N/A]**
Study size	10	Explain how the study size was arrived at **[6]**
Quantitative variables	11	Explain how quantitative variables were handled in the analyses. If applicable, describe which groupings were chosen and why **[6]**
Statistical methods	12	(*a*) Describe all statistical methods, including those used to control for confounding **[7]**
		(*b*) Describe any methods used to examine subgroups and interactions **[7]**
		(*c*) Explain how missing data were addressed **[N/A]**
		(*d*) *Cohort study*—If applicable, explain how loss to follow‐up was addressed **[N/A]** *Case–control study*—If applicable, explain how matching of cases and controls was addressed **[N/A]** *Cross‐sectional study*—If applicable, describe analytical methods taking account of sampling strategy **[7]**
		(*e*) Describe any sensitivity analyses **[N/A]**
Results		
Participants	13^a^	(a) Report numbers of individuals at each stage of study—e.g. numbers potentially eligible, examined for eligibility, confirmed eligible, included in the study, completing follow‐up and analysed **[8]**
		(b) Give reasons for non‐participation at each stage **[N/A]**
		(c) Consider use of a flow diagram **[N/A]**
Descriptive data	14^a^	(a) Give characteristics of study participants (e.g. demographic, clinical, social) and information on exposures and potential confounders **[8–9]**
		(b) Indicate number of participants with missing data for each variable of interest **[8–9]**
		(c) *Cohort study*—Summarize follow‐up time (e.g. average and total amount) **[N/A]**
Outcome data	15^a^	*Cohort study*—Report numbers of outcome events or summary measures over time **[N/A]**
		*Case–control study—*Report numbers in each exposure category, or summary measures of exposure **[N/A]**
		*Cross‐sectional study—*Report numbers of outcome events or summary measures **[8–9]**
Main results	16	(*a*) Give unadjusted estimates and, if applicable, confounder‐adjusted estimates and their precision (e.g. 95% confidence interval). Make clear which confounders were adjusted for and why they were included **[8–9]**
		(*b*) Report category boundaries when continuous variables were categorized **[8–9]**
		(*c*) If relevant, consider translating estimates of relative risk into absolute risk for a meaningful time period **[N/A]**
Other analyses	17	Report other analyses done—e.g. analyses of subgroups and interactions, and sensitivity analyses **[N/A]**
Discussion		
Key results	18	Summarize key results with reference to study objectives **[9–12]**
Limitations	19	Discuss limitations of the study, taking into account sources of potential bias or imprecision. Discuss both direction and magnitude of any potential bias **[13]**
Interpretation	20	Give a cautious overall interpretation of results considering objectives, limitations, multiplicity of analyses, results from similar studies, and other relevant evidence **[9–13]**
Generalisability	21	Discuss the generalisability (external validity) of the study results **[9–13]**
Other information		
Funding	22	Give the source of funding and the role of the funders for the present study and, if applicable, for the original study on which the present article is based **[Title page]**

*Note*: An Explanation and Elaboration article discusses each checklist item and gives methodological background and published examples of transparent reporting. The STROBE checklist is best used in conjunction with this article (freely available on the Web sites of PLoS Medicine at http://www.plosmedicine.org/, Annals of Internal Medicine at http://www.annals.org/, and Epidemiology at http://www.epidem.com/). Information on the STROBE Initiative is available at www.strobe‐statement.org.

Once you have completed this checklist, please save a copy and upload it as part of your submission. When requested to do so as part of the upload process, please select the file type: *Checklist*. You will NOT be able to proceed with submission unless the checklist has been uploaded. Please DO NOT include this checklist as part of the main manuscript document. It must be uploaded as a separate file.

^a^ Give information separately for cases and controls in case–control studies and, if applicable, for exposed and unexposed groups in cohort and cross‐sectional studies.

## APPENDIX B

**TABLE A2:** Adoption of HPCCI sub‐scales from the existing valid and reliable tools

Instrument (reference)	Items	Cronbach' Alpha	Sub‐scales of HPCCI	Items
The EHTAN Questionnaire Tool (Cowan et al., 2008)	108	0.95–0.97	Communication	10
			Teamwork and collaboration	7
			Personal and professional development	8
Advanced practice nurse competency assessment instrument (Sastre‐Fullana et al., 2017)	54	0.84–0.92	Quality improvement	4
			Research and EBP	8
Competency inventory for registered nurse (Liu et al., 2007)	58	0.79–0.86	Leadership and management	10
			Ethical and legal practice	8
			Patient‐centred care	10
Assessment competency list (Tromp et al., 2012)	37	0.89–0.94	Professionalism	6
Patient safety competency self‐evaluation tool (Lee et al., 2014)	41	0.91	Safety	7
Nursing informatics competencies (Honey et al., 2017).	3	‐	Health information technology	3
